# Proteomic analysis of plasma membrane and secretory vesicles from human neutrophils

**DOI:** 10.1186/1477-5956-5-12

**Published:** 2007-08-10

**Authors:** Deepa Jethwaney, Md Rafiqul Islam, Kevin G Leidal, Daniel Beltran-Valero de Bernabe, Kevin P Campbell, William M Nauseef, Bradford W Gibson

**Affiliations:** 1 Buck Institute for Age Research, Novato, CA 94945, USA; 2 Inflammation Program, Department of Medicine, University of Iowa and Veterans Administration Medical Center, Iowa City, IA 52240, USA; 3 Howard Hughes Medical Institute, Senator Paul D. Wellstone Muscular Dystrophy Cooperative Research Center, Department of Molecular Physiology and Biophysics, Department of Neurology, andDepartment of Internal Medicine, University of Iowa, Iowa City, IA 52240, USA

## Abstract

**Background:**

Polymorphonuclear neutrophils (PMN) constitute an essential cellular component of innate host defense against microbial invasion and exhibit a wide array of responses both to particulate and soluble stimuli. As the cells recruited earliest during acute inflammation, PMN respond rapidly and release a variety of potent cytotoxic agents within minutes of exposure to microbes or their products. PMN rely on the redistribution of functionally important proteins, from intracellular compartments to the plasma membrane and phagosome, as the means by which to respond quickly. To determine the range of membrane proteins available for rapid recruitment during PMN activation, we analyzed the proteins in subcellular fractions enriched for plasma membrane and secretory vesicles recovered from the light membrane fraction of resting PMN after Percoll gradient centrifugation and free-flow electrophoresis purification using mass spectrometry-based proteomics methods.

**Results:**

To identify the proteins light membrane fractions enriched for plasma membrane vesicles and secretory vesicles, we employed a proteomic approach, first using MALDI-TOF (peptide mass fingerprinting) and then by HPLC-MS/MS using a 3D ion trap mass spectrometer to analyze the two vesicle populations from resting PMN. We identified several proteins that are functionally important but had not previously been recovered in PMN secretory vesicles. Two such proteins, 5-lipoxygenase-activating protein (FLAP) and dysferlin were further validated by immunoblot analysis.

**Conclusion:**

Our data demonstrate the broad array of proteins present in secretory vesicles that provides the PMN with the capacity for remarkable and rapid reorganization of its plasma membrane after exposure to proinflammatory agents or stimuli.

## Background

Human polymorphonuclear leukocytes (neutrophils or PMN) are essential for optimal host defense against invading microorganisms and employ both oxygen-dependent and -independent agents in concert to kill and degrade ingested microbe [1]. The cell biology of PMN is especially tailored to mediate the rapid and efficient responses that characterize the innate immune system early in inflammation. Stimulation of PMN triggers several concurrent events that together mount a potent cytotoxic response to invading microbes or other noxious agents [2].

The initiation of phagocytosis stimulates the assembly and activation of the NADPH oxidase (reviewed in [3]), resulting in the PMN undergoing a burst of oxygen consumption and generation of reactive oxygen species. The NADPH oxidase is a multicomponent enzyme complex that is unassembled and inactive in the resting PMN, with essential components segregated in distinct cellular compartments (*i.e*. membrane vs. cytoplasm) in the unstimulated cell. When PMN are stimulated, the cytosolic elements translocate to the plasma or phagosomal membrane where they associate with the membrane-bound flavocytochrome b_558 _to form a functional oxidase complex. Simultaneously the intracellular granules fuse with the phagosomal membrane, thereby releasing their contents into the same compartment as that in which the reactive oxygen species are being generated [4-6]. The granule contents include proteolytic enzymes such as elastase [7] proteins that are directly toxic to target microbes such as the defensins [8,9] or bactericidal permeability increasing protein [10], and proteins that convert H_2_O_2 _into more potent antimicrobial species [11]. Reactive oxygen species, antimicrobial proteins, and hydrolytic enzymes not only act independently but also cooperate synergistically to create an environment within the phagosome that is extremely inhospitable to the ingested microbe. Both oxidase assembly and degranulation represent agonist-dependent redistribution of prefabricated biological elements, a strategy of cellular response that is especially tailored to the physiologic responsibilities of PMN within the context of innate immunity and distinctly different from one dependent on transcriptional control of the production of reactive molecules [12].

Recent interest has focused on identification of the various types of granules in PMN and their sequential mobilization during activation. In addition to the distinct granule populations, PMN contain secretory vesicles, a unique and easily mobilizable compartment that co-sediments with plasma membrane in the light membrane fraction of resting PMN [13]. Whereas the lumen of secretory vesicles houses plasma proteins such as human serum albumin, the membranes of this intracellular compartment contain a variety of functionally important membrane proteins [reviewed in [14]]. During exposure to proinflammatory stimuli, the secretory vesicles readily fuse with the plasma membrane, thereby integrating its resident membrane proteins with those constitutively present at the PMN surface [14]. In this way the fusion of secretory vesicles with the plasma membrane transforms the resting PMN to a cell more suited to deliver cytotoxic agents against invading microbes or other threatening noxious agents [15,16].

The purpose of the present study is to employ proteomic analysis of plasma membrane and secretory vesicles from resting human PMN in order to define the repertoire of functionally important membrane proteins available in secretory vesicles for rapid recruitment to the plasma membrane during PMN activation.

## Results

### Resolution of plasma membrane-enriched fractions from resting PMN

The light membrane fraction recovered from a two-step Percoll density gradient separation of cavitated resting PMN [13], the γ fraction, is enriched for plasma membrane vesicles (PMV) but also contains secretory vesicles (SV), a labile intracellular compartment whose membranes contain several functionally important proteins [17]. In light of the lability of SV and the facility with which they fuse with the plasma membrane, it was essential to be confident that PMN used for study were truly at rest. In the absence of endotoxin contamination, PMN isolated from heparinized venous blood using sequential dextran sedimentation and differential density centrifugation on Hypaque-Ficoll are neither primed nor stimulated: they do not consume oxygen, indicating that the NADPH oxidase is neither assembled nor active, and their intracellular compartments remain intact [1]. For our studies, we routinely screen the status of NADPH oxidase activity, using superoxide dismutase-inhibitable reduction of ferricytochrome C to quantitate oxidant production [18]. Routinely, PMN isolated by sequential dextran sedimentation and differential density centrifugation on Hypaque-Ficoll generate 1.01 ± 0.21 nmoles superoxide anion/10^6 ^PMN/10 min (n = 9), whereas PMN stimulated with 100 ng/ml of phorbol myristate acetate produce 78.47 ± 2.48 nmoles superoxide anion/10^6 ^PMN/10 min (n = 9). Using the absence of oxidase activity as a criterion, PMN used in these studies were at rest. Another feature of resting PMN is the presence of 85% of the flavocytochrome b_558 _(a heterodimer of gp91^*phox *^and p22^*phox *^and the membrane component of the phagocyte NADPH oxidase) in the specific granules [19]. To assess the distribution of flavocytochrome b_558 _in PMN used in our studies, we immunoblotted an equal number of cell equivalents of specific granules, PMV, and SV, the subcellular compartments in which gp91^*phox *^is expressed [17]. Consistent with previous reports, the majority of gp91^*phox *^was detected in specific granules (~80%), with PMV and SV expressing ~20% of the remaining total (see Figure A1, additional file 1). Taken together, the absence of oxidase activity and the predominantly intracellular location of flavocytochrome b_558 _demonstrate that the PMN used for study were judged to be in the resting state.

The yield of light membranes retrieved from resting PMN was reproducible, 0.90 ± 0.12 μg protein/10^6 ^cell equivalents (CE) (n = 19). To identify the protein components of secretory vesicles that would be newly available in the plasma membrane after their fusion at the cell surface, we resolved plasma membrane vesicles from secretory vesicles in the membranes of resting PMN using free-flow electrophoresis (FFE).

**Figure 1:**
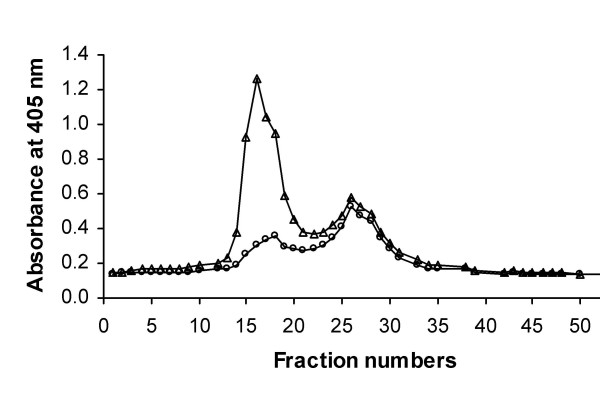
**Separation of plasma membrane vesicles and secretory vesicles from resting PMN**. Isolated resting PMN were disrupted by N_2 _cavitation and fractionated using a two-step discontinuous gradient of Percoll. The γ-band containing the light membranes was recovered, treated with neuraminidase, and subjected to free-flow electrophoresis to resolve plasma membranes vesicles from secretory vesicles. Fractions (96) were collected and assayed spectrophotometrically for alkaline phosphatase activity in the absence (○) and presence (△) of Triton X-100. Data are expressed as units of absorbance at 405 nm. Latent alkaline phosphatase activity indicates the presence of secretory vesicles.

The secretory vesicles were distinguished from the plasma membrane-derived vesicles by the presence of latent alkaline phosphatase activity in the former, detected only after their solubilization in T×100 (Figure 1). Whereas there were two peaks of alkaline phosphatase activity recovered from fractions after FFE, only one peak (fractions 12–20) in resting PMN demonstrated latent activity (Figure 1). The activity of the second peak (fractions 22–28) was unchanged by T×100 treatment, consistent with these fractions representing plasma membrane.

**Figure 2:**
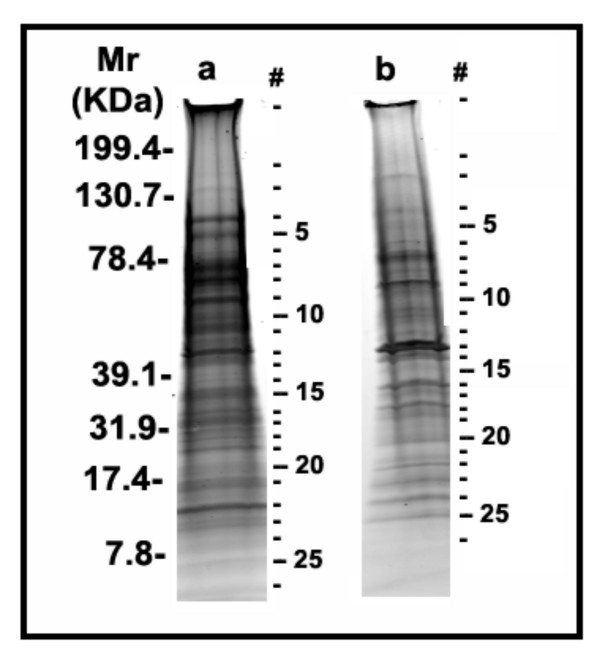
**Proteins in fractions enriched for plasma membrane vesicles or secretory vesicles from resting PMN**. Secretory vesicles (**a**) and Plasma membrane vesicles (**b**) recovered by free-flow electrophoresis of isolated γ fraction from resting PMN were separated by PAGE on 4–20% SDS gradient gel and visualized by SYPRO ruby staining. The bands (indicated by numbers) from top to bottom in each lane were excised from the gel with the help of 1.5 mm band picker and processed with an automatic in-gel digester robot, ProGest as described in Materials and Methods. The numbers assigned to the bands in the gel correspond to the proteins listed in Table A1 (Additional file 2).

In order to minimize potential cross-contamination between the two peaks, we pooled only the centermost fractions of each of the two peaks, sacrificing yield for purity. Using this more restricted collection of vesicles from FFE, we obtained 0.10 ± 0.04 μg protein/10^6 ^CE and 0.14 ± 0.02 μg protein/10^6 ^CE for PMV and SV, respectively (n = 19). Selected fractions from the two peaks were pooled and the component proteins separated by SDS-PAGE and stained with Sypro Ruby (Figure 2) for subsequent excision and analysis. Based on densitometer scanning of both gel lanes, the total protein loaded from the SV-enriched fraction was ~2-fold higher than that in the PMV-enriched material.

### Evidence for other light organelles in PMV- and SV-enriched fractions

**Figure 3:**
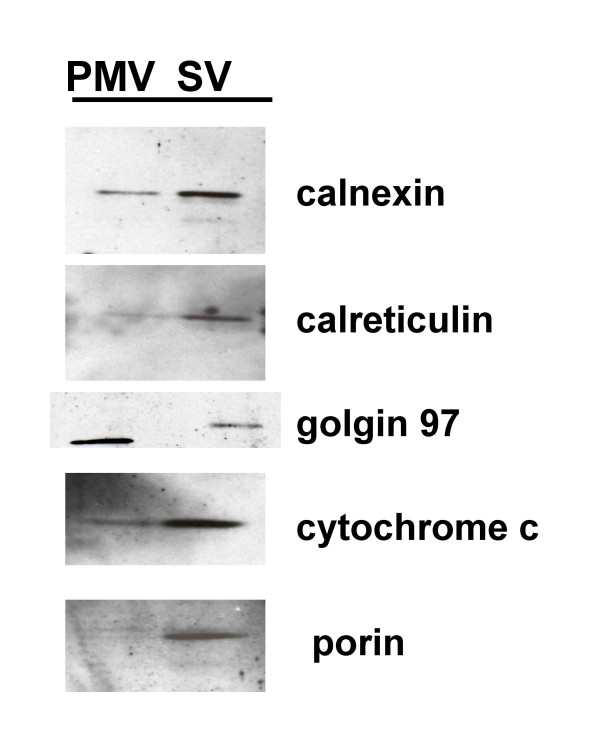
**Immunochemical analysis of plasma membrane vesicle- and secretory vesicle-enriched fractions for other cell organelles**. An equal number of cell equivalents of plasma membrane vesicles (PMV), and secretory vesicles SV, were separated by SDS-PAGE, electroblotted, and probed with antibodies against calreticulin and calnexin (both molecular chaperones residing in the ER), golgin 97 (marker for Golgi), porin and cytochrome c (both markers of mitochondria).

One would anticipate that the light membrane fraction of resting PMN might include not only the PMV and SV, but also membranes from other intracellular organelles with similar low density. Although mature PMN are terminally differentiated and exhibit limited proteins synthesis under resting conditions [1], proteomic analyses of PMN granules [20] and of PMN phagosomes [21] have reported the recovery of proteins selectively expressed in ER, Golgi, and mitochondria. However, there are no published data that directly assess the presence of ER, mitochondria, or Golgi in SV or PMV preparations derived from FFE of resting human PMN. To address this issue, we immunoblotted equal numbers of cell equivalents of plasma PMV and SV isolated by FFE of light membranes recovered from resting PMN, and probed the fractions with antibodies against calreticulin and calnexin (both molecular chaperones residing in the ER), porin and cytochrome c (both markers of mitochondria), and golgin 97 (marker for Golgi) (Figure 3). As anticipated, the aforementioned organelles co-sedimented with SV and with PM. Whereas marker proteins for ER and mitochondria indicated relatively more of these organelles in SV-enriched fractions, an observation that is consistent with the mass spectrometric identification of mitochondrial and ER proteins mentioned above, Golgi membranes co-sedimented with PMV. We interpret the recovery of ER, mitochondrial, and Golgi proteins in these fractions as evidence for co-sedimentation of these organelles with PMV or SV, rather than the *bona fide *expression of the marker proteins in PMV or SV.

### Composition of plasma membrane and secretory vesicle enriched fractions from PMN

To survey the most abundant proteins of plasma membrane vesicles and secretory vesicles, we employed a proteomic approach, first using immunochemistry, and then mass spectrometry (MALDI-TOF and HPLC-MS/MS) to analyze the two vesicle populations from resting PMNs after separation by 1D gel electrophoresis. Due to the complexity of these mixtures, even after 1D gel separation, MALDI-TOF peptide mass fingerprinting of the 27 gel slices from each of the two preparations identified only a few of the most abundant proteins: integrin alpha-M and matrix metalloproteinase-9 in the secretory vesicle preparation, and moesin and beta-actin in the plasma membrane fraction (data not shown). To obtain a more in-depth coverage of the proteins in these two preparations, the same trypsin-digested gel slices where subjected to HPLC-MS/MS analysis. In this latter case, 43 and 37 unique proteins could be identified in the vesicle and plasma membrane fractions, respectively (see additional file 2, for Table A1).

**Figure 4:**
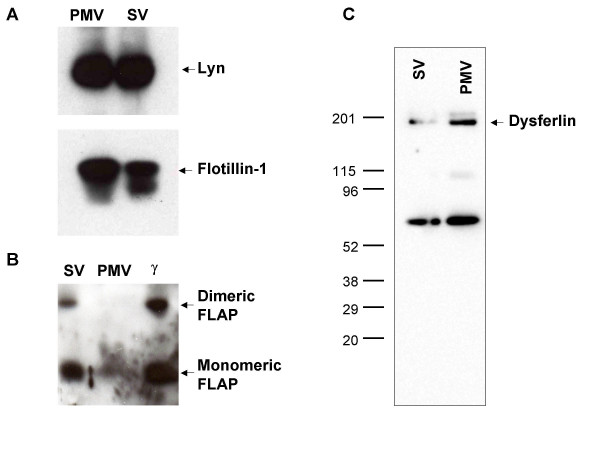
**Immunochemical analysis of specific proteins in PMV- and SV-enriched fractions**. Plasma membrane vesicles (PMV) and secretory vesicles (SV) fractions were separated by SDS-PAGE, electroblotted and probed with antibodies against several proteins: (**A**) Lyn and Flotillin, (**B) **FLAP, and **(C) **Dysferlin. The lower molecular weight band visualized in panel C of both lanes does not correspond to the expected size for dysferlin and may be the result of limited proteolysis of dysferlin during organelle isolation or a non-specific immunoreactive protein unrelated to dysferlin.

Many proteins were recovered from both compartments. For example, lyn and flotillin-1, markers of detergent-resistant membranes or lipid rafts [22-24], were equally distributed in plasma membrane vesicles and secretory vesicles, based on Western blot analysis (Figure 4A). More comprehensive analysis by HPLC-MS/MS likewise demonstrated that the two compartments shared many of the same proteins or protein classes, most notably those that participate in adhesion, cytoskeletal events, and signal transduction (Table 1 and additional file 3 for Table A2,). In many of these cases, these proteins have been previously identified in vesicle compartments, such as the beta integrins, CD13, CD45, flavocytochrome b_558_, and Rabs. Although these mass spectrometric data sets were not quantitative, one can use spectral counts as a rough indicator of relative protein abundances [25]. It should be noted, however, that the total spectral counts obtained for all proteins in the enriched SV preparation was ~2.6 times higher than that obtained from the PMV preparation, consistent with the densitometry estimation of a 2-fold difference in total protein loaded onto each gel, and therefore should also be considered when comparing these fractions. For example, additional file 3 (Table A2) provides spectral count information for the eight proteins identified by HPLC MS/MS, and indicates that some of these were roughly similar in concentration, i.e., guanine nucleotide binding protein G (i) alpha-2 subunit, CD18, and Beta-actin. In contrast, CD11b, matrix metalloproteinase-9, lactoferrin, myeloperoxidase and serum albumin were all significantly more abundant in the enriched secretory vesicle fraction, even when accounting for total protein loading differences (Figure 2). As previously reported, the secretory vesicles also contained serum albumin [26], although its presence in the enriched PMV fraction has not been reported before and appears to be at considerably lower concentrations.

**Table 1:** Classification of proteins identified from fractions enriched for plasma membrane and secretory vesicles.

***Plasma membrane vesicles***	***Secretory vesicles***
**Adhesion**	

Integrin alpha-M (CD11b) Integrin beta-2 (CD18 antigen) Integrin alpha-IIb Intercellular adhesion molecule-3 (ICAM-3) Phagocytic glycoprotein I (CD44 antigen)	Integrin alpha-M (CD11b) Integrin beta-2 (CD18 antigen) ADP-ribosyl cyclase 2 (CD157 antigen) Erythrocyte band 7 integral membrane protein (Stomatin)

**Cytoskeletal**	

Beta-actin (ACTB) Alpha-actinin 1 Alpha-actinin 4 Cofilin, non-muscle form Coronin-1A Moesin Myosin light polypeptide 6 Myosin regulatory light chain 2, nonsarcomeric Tropomyosin alpha 3 chain Tropomyosin beta chain	Beta-actin (ACTB) Myosin-9 Tubulin alpha-ubiquitous chain

**Signal transduction**	

Guanine nucleotide-binding protein G(i), alpha-2 subunit Tyrosine protein kinase Lyn * Flotillin* B-cell receptor-associated protein 31 Chloride intracellular channel protein 1 Guanine nucleotide-binding protein G(I)/G(S)/G(T) beta subunit 1 HLA class I histocompatibility antigen, A-26 alpha chain Interferon-induced transmembrane protein 1 Ras-related protein Rap-1A Ras-related protein Rab-5A Ras-related protein Rap-1b Ras-related protein Rab-27B Synaptosomal-associated protein 23	Guanine nucleotide-binding protein G(i), alpha-2 subunit Tyrosine protein kinase Lyn * Flotillin* Adipocyte plasma membrane-associated protein C5a anaphylatoxin chemotactic receptor Dysferlin Leukocyte surface antigen CD47 5-lipoxygenase activating protein (FLAP) Solute carrier family 2, facilitated glucose transporter, member 3

**NADPH oxidase**	

*Cytochrome b-245 heavy chain (gp91*^*phox*^)*	Cytochrome b-245 heavy chain (gp91^phox^)

**Differentiation**	

	Myeloid-associated differentiation marker (SB135) Leukocyte common antigen (CD45 antigen)

**Serum protein**	

Serum albumin	Serum albumin Alpha-1-antitrypsin Ig gamma-1 chain C region

**Mitochondrial/Microsomal**	

	ATP synthase alpha chain, mitochondrial ATP synthase beta chain, mitochondrial Citrate synthase, mitochondrial Cytochrome P450 4F2 60 kDa heat shock protein, mitochondrial (Hsp60) Isocitrate dehydrogenase [NADP], mitochondrial Malate dehydrogenase, mitochondrial Sarcoplasmic/endoplasmic reticulum calcium ATPase 3 Sulfide: quinone oxidoreductase, mitochondrial Trifunctional enzyme beta subunit, mitochondrial Vacuolar ATP synthase subunit d

**Metabolic**	

	Aldehyde dehydrogenase 3B2 Dehydrogenase/reductase SDR family member 7 Tyrosine-protein phosphatase non-receptor type substrate 1

**Granule**	

Lactoferrin Matrix metalloproteinase 9 Myeloperoxidase Cathepsin G Azurocidin	Lactoferrin Matrix metalloproteinase 9 Myeloperoxidase Aminopeptidase N (CD13 antigen) Eosinophil peroxidase

**Cytosolic**	

14-3-3 protein zeta/delta	Dolichyl-diphosphooligosaccharide-protein glycosyltransferase 48 kDa subunit

**Unknown**	

Actin-related protein 2 Golgi-associated plant pathogenesis related protein 1 Protein FAM49B Tetratricopeptide repeat protein 10	ERO1-like protein alpha Pantophysin

* Proteins identified only by immunoblotting are listed in *italics*. All other proteins identified by mass spectrometry.

In other cases, there were marked differences in classes of proteins identified between the plasma membrane and secretory vesicles. For example, several mitochondrial and ER proteins (11), metabolic enzymes (3), NADPH oxidases (1), and proteins involved in differentiation (2) were identified by mass spectrometry only in the secretory vesicle preparation. The presence of so many mitochondrial and ER proteins in the secretory vesicle preparation was not unexpected in the light membranes from eukaryotic cells, although mature PMN are terminally differentiated and exhibit limited proteins synthesis under resting condition [1]. Electron microscopy analysis of the two vesicle preparations also identified mitochondria and ER organelles in the SV fraction (data not shown).

In addition to the previously recognized proteins, several novel proteins were identified that had not previously been demonstrated to reside in secretory vesicles including 5-lipoxygenase-activating protein (FLAP) and dysferlin. Whereas FLAP had previously been recovered from PMN [27], dysferlin had been identified by a proteomic analysis to be located in the peroxidase-negative granules of resting PMN [20]**: **neither protein was previously reported to be in PMV or SV.

**Figure 5:**
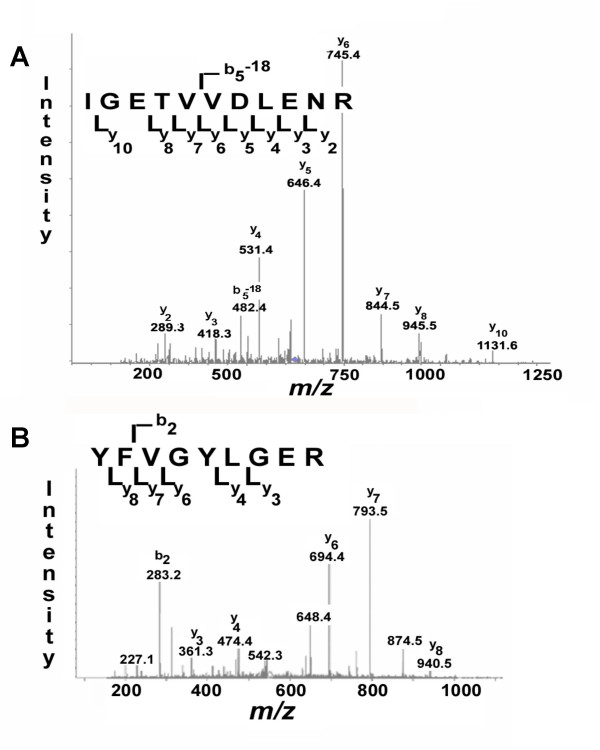
**Tandem mass spectra for FLAP and dysferlin**. **(A) **The MS/MS spectrum of dysferlin peptide "IGETVVDLENR" of m/z 623.5 (Molecular mass of 1245.4 Da) after free flow electrophoresis, tryptic digestion and 1D gel (spot 9 in Figure 2, SV) of the secretory vesicles and **(B) **The MS/MS spectrum of FLAP peptide "YFVGYLGER" of m/z 552.3 (molecular mass of 1104.2 Da) after free flow electrophoresis, tryptic digest and ID gel (Spot 1 on Fig 2, SV) of the secretory vesicles.

In order to validate the novel identification of FLAP and dysferlin using an independent analytical method, PMV and SV were probed immunochemically for the presence of FLAP (Figure 4B) and dysferlin (Figure 4C). Both monomeric (18-kDa) and dimeric FLAP were detected immunochemically in PMN membranes, with most of the FLAP in the SV and relatively little present in the PMV (Figure 4B). Likewise, the presence of dysferlin in SV and PMV from resting PMN was confirmed (Figure 4C). In addition the identities of dysferlin (Figure 5A) and FLAP (Figure 5B) were confirmed by MS/MS spectra of specific tryptic peptide fragments.

### Secretogogue-induced redistribution of dysferlin

**Figure 6:**
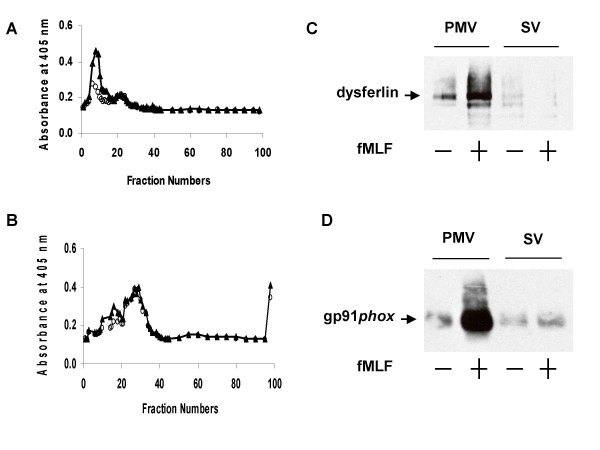
**Secretogogue-induced redistribution of dysferlin**. **(A) **Isolated resting PMN were disrupted by N_2 _cavitation and fractionated using a two-step discontinuous gradient of Percoll. The γ band containing the light membranes was recovered, treated with neuraminidase, and subjected to free-flow electrophoresis to resolve plasma membranes vesicles from secretory vesicles. Fractions (96) were collected and assayed spectrophotometrically for alkaline phosphatase activity in the absence (○) and presence (△) of Triton X-100. **(B) **The PMN were isolated as above and were exposed to fMLF (formyl methionyl-leucyl-phenylalanine). Fractions (96) were collected and assayed spectrophotometrically for alkaline phosphatase activity in the absence of (○) and presence (△) of Triton X-100 after exposure to fMLF. The exposure to fMLF resulted in a loss of SV (*i.e*. loss of latent alkaline phosphatase activity), consistent with their fusion with the plasma membrane. **(C) **Purified PMV and SV from resting or fMLF-stimulated PMN were separated by SDS-PAGE, electroblotted, and the resulting blots probed with anti-dysferlin. **(D) **As a control for intracellular membrane recruitment, samples were also probed with 54.1, as flavocytochrome b_558 _expression at the PMN surface increases with agonist-stimulated granule and secretory vesicle fusion with plasma membrane.

Given our demonstration that lights membranes from ER, Golgi, and mitochondria were present in our PMV- and SV-enriched fractions, we reasoned that our recovery of dysferlin in the SV fraction could reflect the *bona fide *presence of dysferlin SV or a contribution from contaminating light membranes that co-localized with SV after FFE. To resolve between these two possibilities, we subjected light membranes from resting and stimulated PMN to FFE to determine if secretogogue treatment elicited a redistribution of dysferlin to PMV. PMN were stimulated with 10 μM formyl-methionyl-leucyl-phenylalanine (fMLF), a well characterized PMN secretogogue, for 15 minutes at 37°C and PMV and SV were isolated and analyzed. As demonstrated by the redistribution of latent alkaline phosphatase activity, exposure to fMLF resulted in a disappearance of SV, manifested as the loss of latent alkaline phosphatase activity, and an increase in the non-latent activity (Figures 6A and 6B), consistent with fusion of SV with the PMV. Purified PMV and SV from resting or fMLF-stimulated PMN were separated by SDS-PAGE, electroblotted, and the resulting blots probed with anti-dysferlin (Figure 6C). As a control for intracellular membrane recruitment, samples were also probed with 54.1, as flavocytochrome b_558 _expression at the PMN surface increases with agonist-stimulated granule and secretory vesicle fusion with PMV (Figure 6D) [13]. Dysferlin expression at the cell surface increased after secretogogue treatment, just as did flavocytochrome b_558 _expression. These data indicate that, like flavocytochrome b_558_, dysferlin is recruited from intracellular vesicles to fuse at the PMV and that the dysferlin detected in resting PMN was in SV and not due to contamination with other light membrane organelles.

## Discussion

Agonist-dependent PMN stimulation during acute inflammation, including activation of the NADPH oxidase and release of granule contents, demonstrates the efficient and speedy manner in which innate immunity up-regulates its machinery in response to microbial threats [28]. Both primed as well as fully activated PMN increase surface expression of a wide variety of receptors and functionally important molecules by recruitment from intracellular stores [1], which include not only the specific and azurophilic granules [4] but also membrane-bound secretory vesicles [17]. In these studies we employed proteomic analysis to survey the proteins present in plasma membrane and in the extremely labile secretory vesicle pool to better understand the complete repertoire of functional remodeling that can accompany secretory vesicle fusion.

Secretory vesicles are a relatively recently identified subcellular compartment in PMN, separated by free-flow electrophoresis of the light membrane fraction isolated from discontinuous Percoll gradients [17,29]. Although a method using sucrose flotation also has been employed to recover SV [30,31] it has never been compared with FFE. However, features of the fractions recovered by sucrose flotation closely parallel those of the secretory vesicles isolated by FFE, suggesting that the method may well recover authentic SV. Whereas the lumen of the secretory vesicles contains plasma proteins, its membranes possess flavocytochrome b_558_, β_2 _integrin CD11b/18, formyl peptide receptor, CR1, CD16, and leukosylin [17,26,32-34]. Proinflammatory stimuli mobilize secretory vesicles and trigger graded degranulation of the specific granules, thereby integrating membrane proteins from these intracellular vesicles and granules into the PM. Consequently, the resting PMN becomes transformed into a cell more responsive to subsequent challenge by increasing the number of effectors molecules available at the PMN surface [14].

Of the 43 proteins recovered from secretory vesicles, a minority had been identified previously in this neutrophil compartment. The β_2 _integrins CD11b and CD18, CD13, CD45, flavocytochrome b_558_, V-type H^+ ^ATPase, and the Rabs are functionally important proteins that were previously identified as constituents of membranes of secretory vesicles [14,35]. For most proteins recovered however, this report represents their first direct identification in human PMN or in PMN secretory vesicles. Highly expressed on myeloid cells throughout all stages of differentiation [36], CD157 is detected on the surface of mature PMN and increases after exposure to formyl peptides [37]. CD157 is a glycosylphosphatidyl inositol-anchored protein that has been implicated in inducing cytoskeletal rearrangement important for shape changes integral for PMN adhesion and movement [37]. Like many other functionally important membrane proteins in PMN, CD157 is thus compartmentalized in secretory vesicles as an intracellular reservoir easily recruited during PMN activation. Proteins such as cofilin [38,39], CD13 [40], stomatin [41,42], Rab5 [43], and development- and differentiation-enhancing factor 2 [44] have been implicated in endosomal pathway recycling or other events in phagosome maturation [45] in a variety of cell types.

Proteomic analysis is extremely sensitive, demonstrated in our studies by the detection of two classes of contaminants in our PMV- and SV-enriched fractions. We identified resident proteins specific for ER, Golgi, and mitochondria; although the presence of so many mitochondrial and ER proteins is not unexpected in the light membranes from eukaryotic cells, many human PMN are terminally differentiated and exhibit very limited protein synthesis under resting conditions [1] making such proteins at extremely low abundance. As we noted, our analysis also included the identification of granule-associated proteins in fractions that are free of granules. Granules are partially disrupted during the N_2 _cavitation, as observed in the original report of the method (reference [13]) and on several occasions since that publication [46,47]. In a recent report of proteomic analysis of the granules from human PMN [20], the authors documented the release of soluble granule proteins during sample preparation with resultant cross contamination of the three granule populations subsequently analyzed. Most (75%) of the gelatinase was recovered in the gelatinase-positive granules, but 20% and 5% of the total contaminated specific and azurophilic granules, respectively. By the same token, most (73%) of the myeloperoxidase was recovered in MPO-containing azurophilic granules, but 20% and 7% of the total contaminated specific and gelatinase-positive granules, respectively. Thus, soluble granule proteins are released to a limited degree during sample preparation and can contaminate other fractions. We believe that this phenomenon explains our detection of granule proteins in granule-free fractions. It is likely that the soluble proteins released from the granules associate with vesicular membranes and thereby co-sediment in the particular membrane fraction in which they were recovered. Consistent with this interpretation, most of the PMN granule proteins, including myeloperoxidase, azurocidin, lactoferrin, and cathepsin G, as well as the eosinophil-derived eosinophil peroxidase, are present in millimolar concentrations, are extremely cationic, and avidly associate with membranes. Furthermore, PMV and SV-enriched fractions also contained actin and actin-associated proteins, which very likely reflect cytoskeletal contamination of the fractions. The challenges posed by the overabundance of cationic granule proteins and cytoskeletal elements to obtaining pure preparations of subcellular membrane-bound compartments can be decreased, in part, by washing membranes with carbonate buffers at pH 11, as done recently in a proteomic analysis of granule membrane proteins [24]. Because of concern for the relative lability of PMV and SV in contrast to granules, we elected to study the recovered fractions without washing with relatively harsh conditions. Consequently, PMV- and SV-enriched fractions in our studies were contaminated with soluble granule proteins.

Our identifications of FLAP and dysferlin in SV and PMV of resting PMN represent novel findings. First identified over a decade ago [27], FLAP is an 18-kDa membrane protein that is essential for 5-lipoxygenase activity and therefore for the biosynthesis of leukotrienes [48]. Inhibition of FLAP translocation blocks leukotriene production by stimulated cells, thus intimately linked FLAP redistribution with 5-lipoxygenase activity [49,50]. FLAP is required for the calcium-dependent translocation of 5-lipoxygenase from cytosol to nuclear membrane, a prerequisite for 5-lipoxygenase activity [51,52]. Immunoelectron microscopy of ultra-thin frozen sections of resting and stimulated PMN demonstrates localization of FLAP exclusively on the nuclear membrane; no other cellular compartments, including plasma membrane were immunoreactive [46]. The previous failure to detect FLAP at plasma membrane or in secretory vesicles could reflect technical limitations of the anti-peptide antibody used, as suggested by the authors [53], as it was raised to an 11 amino acid linear region in FLAP [50]. More recently both monomeric and dimeric FLAP were identified in the light membranes recovered from the post-nuclear supernatant of sonicated human PMN [54]. It is possible that the N_2 _cavitation used to disrupt PMN to generate membrane vesicles may have inadvertently resulted in contamination of the starting material with nuclear membranes. However this explanation appears less likely, as the low speed centrifugation of the cavitate that precedes loading the Percoll gradient removes ~85% of the DNA [13] and we recovered no other nuclear protein in our proteomic analysis (Table 1). However, additional studies directly examining the subcellular location of FLAP in resting PMN are needed to resolve this issue.

Notable among the novel proteins identified by HPLC-MS/MS in secretory vesicles is LGMD2B, the membrane protein encoded by the gene that is mutated in two human muscular dystrophies, limb-girdle muscular dystrophy type 2B (LGMD2B) [55,56] and Miyoshi myopathy (MM) [57]. Dysferlin is a member of the newly described ferlin protein family that also includes myoferlin and otoferlin. These proteins share homology with fer-1, a spermatogenesis factor in *C. elegans *[reviewed in [58]]. Mutations in fer-1 compromise vesicle fusion with the plasma membrane, whereas dysferlin functions in the normal repair of the plasma membrane of skeletal muscle [59], observations that suggest that the dysferlin may participate in fusion events at the plasma membrane. Our data demonstrate agonist-dependent redistribution of dysferlin from SV to PMV but do not address how dysferlin might directly contribute to this up-regulation. It is not known if dysferlin mediates directly or cooperates with other proteins to facilitate membrane fusion. It is possible that in PMN dysferlin mediates the fusion of secretory vesicles with plasma membrane during PMN priming in response to proinflammatory stimuli or as part of membrane remodeling that accompanies PMN activation, as seen during adhesion, endothelial transmigration, chemotaxis, and phagocytosis. The involvement of dysferlin in PMN-mediated immune response is supported both by the exuberant inflammatory infiltrate observed in the muscles of patients with LGMD2B and MM, and by histological changes seen in dysferlin knock-out mice. Furthermore, recent reports have noted that PMN depletion has a protective effect in muscular dystrophies. Further characterization of the activity of dysferlin in secretory vesicles may provide important and novel insights into PMN cell biology.

## Conclusion

Our data demonstrate that the broad array of proteins present in secretory vesicles that provides the PMN with the capacity for remarkable and rapid reorganization of its plasma membrane after exposure to proinflammatory agents or stimuli. The increased surface expression of membrane proteins from secretory vesicles coupled with the amplification of various intracellular signaling pathways allow the PMN to rapidly change from a resting state to an activated phenotype better primed for antimicrobial action. Ongoing studies to extend the known repertoire of proteins present in secretory vesicles and their functional consequences may reveal novel insights into the mechanisms of PMN activation during acute inflammation.

## Methods

### Reagents and antibodies

The protease inhibitors [phenylmethylsulfonyl fluoride (PMSF), aprotinin, phosphoramidone, n-tosyl-lysyl-chloromethyl ketone (TLCK), n-tosyl-phenyl-chloromethyl ketone (TPCK), amidinophenylmethylsulfonylfluoride (APMSF), E-64, leupeptin, and pepstatin, diisopropylfluorophosphate (DFP)], neuraminidase type X from *Clostridium perfringens*, and *p*-nitrophenyl phosphate, 2-amino-2-methyl-1-propanol were purchased from Sigma Chemical Co. (St. Louis, MO). Density gradient centrifugation media, Percoll, and Hypaque-Ficoll were purchased from Amersham Biosciences (Uppsala, Sweden). Sodium dodecyl sulphate (SDS) was purchased from Research Products International Corp. (Mt. Prospect, IL); acrylamide was obtained from Bio-Rad laboratories (Hercules, CA); Hanks' balanced salt solution (HBSS) was purchased from Bio Whittaker (Walkersville, Maryland). BCA Protein Assay Kit and ECL Western Blotting Detection Reagents were obtained from Pierce (Rockford, IL). Endotoxin-free saline and H_2_O were purchased from Baxter Healthcare Corporation (Deerfield, IL).

Materials related to proteomics, such as sample buffers and 1D 4–20% PAGE gels were obtained from Bio-Rad Laboratories (Hercules, CA). Gel stain SYPRO ruby was obtained from Molecular Probes/Invitrogen (Carlsbad, CA). For proteolysis, sequencing grade, modified porcine trypsin was purchased from Promega (Madison, WI). Additional reagents for analytical protein chemistry including iodoacetamide and dithiothreitol were obtained from Sigma (St. Louis, MO). HPLC solvents such as acetonitrile and water were obtained from Burdick & Jackson (Muskegon, MI). For MALDI-MS experiments a matrix solution of α-cyano-4-hydroxycinnamic acid in acetonitrile/methanol was purchased from Agilent Technologies (Palo Alto, CA).

Antibody against lyn was obtained from Santa Cruz Biotechnology (Santa Cruz, CA). Antibody against flotillin-1 was purchased from BD Transduction Laboratories (BD Sciences, San Jose, CA). Antibody against dysferlin, NCL-Hamlet, was purchased from Novocastra (Newcastle upon Tyne, UK). Secondary antibodies used: horseradish peroxidase (HRP)-conjugated goat anti-mouse was purchased from Bio-Rad Laboratories (Hercules, CA); HRP-conjugated rabbit anti-goat IgG was purchased from ICN Biochemicals, Inc. (Aurora, OH); and HRP-conjugated donkey anti-rabbit IgG was purchased from Amersham Biochemicals UK limited. Immunoblotting for 5-lipoxygenase activating protein was kindly performed by Dr. Roy J. Soberman (Harvard University, Boston, MA).

All reagents and materials used in the preparation of PMN (*i.e*. HBSS, Hypaque-Ficoll, Dextran/NaCl, H_2_O, and saline solutions) were free of endotoxin at <10 pg/ml, as determined by *Limulus *amebocyte lysate assay (QCL-1000, Bio Whittaker, Inc., Walkersville, MD).

### Neutrophil isolation

Heparinized, venous blood was obtained from healthy individuals after informed consent and in accordance with a protocol approved by the Institutional Review Board for the University of Iowa. Human PMN were isolated by dextran sedimentation followed by Hypaque-Ficoll differential density gradient centrifugation and hypotonic lysis of erythrocytes as described previously [15]. Purified PMN ≥ 95% of the cells of the preparation were resuspended in calcium-free HBSS and kept on ice until use.

### Isolation of PMN plasma membranes

PMN were disrupted by N_2 _cavitation and plasma membrane-rich fractions were isolated by centrifugation of the postnuclear supernatant on a density gradient of Percoll, as previously described [13]. Briefly, PMN were treated with 1 mM DFP at 4°C for 20 min to inactivate serine esterases, washed, and resuspended at 1.5–2.0 × 10^8 ^PMN/ml in 3 ml of relaxation buffer (RB) (10 mM PIPES, 100 mM KCl, 3 mM NaCl, 3.5 mM MgCl_2_, 1 mM ATP, 1.25 mM EGTA, pH 7.3) containing a mix of protease inhibitors (0.5 mM PMSF, 1 μM aprotinin, 1 μM phosphoramidone, 1 μM TLCK, 2 μM TPCK, 1 μM APMSF, 1 μM E-64, 0.5 μM leupeptin, 0. 1 μM pepstatin), and disrupted by N_2 _cavitation (350 psi). All manipulations were carried out at 0–4°C. The cavitate was centrifuged at 200 × *g *for 10 min to remove nuclei and unbroken cells, and 2–3 ml of post nuclear supernatant were loaded onto a 2 step Percoll gradient in RB [13]. Density gradients were generated by centrifugation at 48,400× *g *for 20 min in a JA-20 rotor (Beckman). After centrifugation at 140,000 × *g *for 1 h to remove residual Percoll, the γ fraction, which contains plasma membranes and secretory vesicles, was resuspended in RB containing protease inhibitors, flash frozen in a dry ice-methanol bath, and stored at -80°C.

### Free flow electrophoresis

In order to separate plasma membrane vesicles from secretory vesicles in the γ fraction isolated from the Percoll gradient of capitates PMN, free flow electrophoresis was performed, according to previously published methods [17,60]. To reduce the surface charge of plasma membrane vesicles, the γ fraction was treated with neuraminidase type X from *Clostridium perfringens *(0.02 units/ml) for 30 min at 37°C and recovered by ultra centrifugation (120,000 × *g *for 45 min). The pellet was resuspended in chamber buffer (5 mM triethanolamine, 5 mM acetic acid, 270 mM sucrose, pH 7.4, conductivity: 0.42 mohm^-1^) by 15 aspirations through a 21-gauge syringe and adjusted to a protein concentration of approximately 1 mg/ml. Electrode buffer was 50 mM triethanolamine and 50 mM acetic acid, pH 7.4 and the chamber buffer was degassed before electrophoresis. Free flow electrophoresis was performed using an Elphor HV 600 (Bender & Hobein, Munich, Germany) at 5°C, a sample inlet of 3.0 ml/h, a chamber buffer flow rate of 3.2 ml/h/fraction, and the current set to 100 mA giving a voltage of 1000 V ± 5%, as previously described [17,60]. Fractions (96) were collected and assayed for the presence of alkaline phosphatase activity, using a previously described method. Briefly, 100 μl of each fraction was added to separate wells of a 96-well flat-bottom plate and then mixed with 200 μl of reaction buffer (5 mM *p*-nitrophenyl phosphate in 100 mM 2-amino-2-methyl-1-propanol, pH 10.0). Reaction mixtures were incubated (15 min, 37°C), and the absorbance was then read in a microplate reader at 405 nm. Based on the profile of non-latent alkaline phosphatase activity representing plasma membrane (determined in the absence of Triton X-100) and latent alkaline phosphatase activity representing secretory vesicles (determined in the presence of Triton X-100), selected fractions were pooled and the membranes recovered by centrifugation (100,000 × *g *for 20 h at 4°C in a Beckman SW 32Ti rotor). The membrane pellet was collected and resuspended in RB. To remove residual sucrose, the suspension was centrifuged for 30 min at 100,000 × *g *at 4°C in a Beckman TLA 120.2 rotor. The membrane pellet was resuspended in RB, flash frozen, and stored at 4°C until further use.

### Gel electrophoresis and immunoblotting

Fractions of plasma membrane and secretory vesicles separated by free flow electrophoresis were solubilized in SDS-sample buffer (62 mM Tris-HCl, pH 6.8, 2.3 % SDS, 5 % BME (v/v), 5 % glycerol), boiled for 5 min at 100°C and were run on 4–20% SDS PAGE mini ready gels (Bio-Rad, Hercules, CA) and stained with Sypro Ruby (Molecular Probes, Eugene, OR) according to manufacturer's instructions. The gels were scanned on Typhoon 8610 variable model imager and densitometer analysis were done by ImageQuant5.2 (Molecular Dynamics, part of Amersham Pharmacia Biotech, Piscataway, NJ). For studies of dysferlin, 3–15% gradients gels were used for SDS-PAGE. For immunoblotting, gels were electro transferred to nitrocellulose membranes (Schleicher & Schuell, Florham Park, NJ) at room temperature at constant voltage of 50 volts for 3 hr and blots were processed using the ECL kit according to the manufacturer's instructions.

### In-gel tryptic digestion of proteins

The protein bandsvisualized by SYPRO Ruby staining were labeled from top to bottom ineach lane (see Figure 2), excised using a 1.5 mm gel picker (# P2D1.5, The Gel Company, San Francisco, CA) and processed with an automatic in-gel digester robot, ProGest (Genomic Solutions, Ann Arbor, MI). Processing steps consisted of destaining and dehydrating the excised gel slices with acetonitrile, reduction with 10 mM DTT at 60°C for 30 min, alkylation of cysteine residues with 100 mM iodoacetamide (37°C, 45 min), and proteolytic digestion using 125–250 ng sequencing grade trypsin (Promega, Madison, WI) at 37°C for 4 h. The resulting tryptic peptides were then extracted from the gel by aqueous/10% formic acid extraction and analyzed by mass spectrometry [61].

### Mass spectrometry

Mass spectra of digested gel spots were obtained by matrix-assisted laser desorption ionization time-of-flight (MALDI-TOF) mass spectrometry on a Voyager DESTR plus instrument (Applied Biosystems, Framingham, MA). All mass spectra were acquired in positive-ionization mode with reflectron optics. The instrument was equipped with a 337 nm nitrogen laser and operated under delayed extraction conditions; delay time 190 nsec, grid voltage 66–70% of full acceleration voltage (20–25 kV). All peptide samples were prepared using a matrix solution consisting of 33 mM α-cyano-4-hydroxycinnamic acid in acetonitrile/methanol (1/1; v/v); 1 μl of analyte (0.1–1 pmol of material) was mixed with 1 μl of matrix solution, and then air-dried at room temperature on a stainless steel target. Typically, 50–100 laser shots were used to record each spectrum. The obtained mass spectra were externally calibrated with an equimolar mixture of angiotensin I, ACTH 1–17, ACTH 18–39, and ACTH 7–38.

All proteolytic peptide extracts were analyzed by HPLC MS/MS using an Agilent Nanoflow Proteomics system that consisted of a nanoflow liquid chromatograph (LC) system coupled to an Agilent 1100 Series XCT ion trap mass spectrometer with an on-line orthogonal nanoelectrospray source. For LC separations, samples (~8 μl) were injected and then enriched and de-salted on a trap column (Zorbax 300SBC18, 150 mm × 75 μm, 3.5 μm) before being transferred and separated on a Zorbax 300SB-C18 nanocapillary column (5 mm × 300 μm, 5 μm) with a flow rate of 300 nl/min. The LC gradient consisted of 3% B at 0 min, 3% B at 5 min, 15% at 8 min, 45% at 50 min, 90% at 55 min, 90% at 60 min, 3% at 61 min and a stop time of 75 min. Solvents were: A = 0.1% formic acid in water, B = 0.1% formic acid in acetonitrile In all cases, peptide samples were analyzed in the positive ion mode under the following hardware and software conditions: Vcap: typically 1800–2000 V; Drying gas flow: 5 L/min; Drying gas temperature: 300°C; Skim 1: 30 V; Capillary exit offset: 75 V; Trap Drive: 85; Averages: 2; ICC: On; Maximum accumulation time: 150 ms; Smart Target: 125,000; MS Scan range: 300–2200; Automatic MS/MS: Peptide Scan mode (standard-enhanced for MS and Ultra Scan for MS/MS); Number of parents: 3; Averages: 2; Fragmentation amplitude: 1.3 V; SmartFrag: On, 30–200%; Active Exclusion: On, 2 spectra, 1 min; Prefer +2: on; MS/MS Scan Range: 50–2200 *m/z*.

### Database searches

Mass spectrometric data were analyzed with two in-house licensed bioinformatics database search engine systems, RADARS (Genomic Solutions, Ann Arbor, MI) [62] and Mascot Wizard (Matrix Sciences, London, United Kingdom) [63] MALDI-MS data were analyzed with RADARS and Mascot Wizard using the search engine ProFound and Mascot bioinformatics database search engine for Peptide Mass Fingerprint (PMF) matching against peptides from known protein sequences entered in publicly available SwissProt protein databases using the following parameters: internal calibration using trypsin autolysis masses (*m/z *842.5100 and 2211.1046), 100 ppm mass accuracy, 2 missed proteolytic cleavages allowed. Profound uses an 'expectation value' for data quality control that gets smaller as the probability of a nonrandom (real) protein hit increases, *e.g*. 1 × 10^-2 ^is a 1 in 100 chance of being a random hit (confidence > 99.0%), 1 × 10^-3 ^is a 1 in 1000 chance of being a random hit (confidence > 99.9%); protein matches are considered significant for scores with expectation value <5 × 10^-2 ^(confidence >95%)[62]. Mascot Wizard score was -10*Log(P), where P is the probability that the observed match was a random event. Protein scores greater than 64 are significant (p < 0.05). For Ion-Trap-MS/MS data sets spectra were submitted to Mascot searching against the publicly available SwissProt database for *Homo sapiens*. Mascot uses a probability based 'Mowse Score' to evaluate data obtained from tandem mass spectra, e.g. for a score > 42, protein matches are considered significant [63]. For LC-MS/MS acquired data a minimum of two observed peptides that were selected for tandem mass spectrometry was required to confirm protein identification; in the few cases where only one unique peptide per protein was selected for MS/MS, the MS/MS spectrum inspected "manually" based on previously published criteria and thus confirmed or deleted from the identification list [64]. These latter proteins are listed in *italics *in table A1 (Additional file 2). All "single hit" proteins listed in table A1 (additional file 2) were additionally validated by comparing MS/MS spectra to those in the global proteome machine (GPM). Details of these methods and inclusion criteria are described more fully in the table A1 (additional file 2) legend section.

## Abbreviations

DFP, Diisopropylfluorophosphate; ECL, Enhanced chemiluminescence; fMLF, formyl methionyl-leucyl-phenylalanine; FFE, free-flow electrophoresis; FLAP, 5-lipoxygenase-activating protein; HBSS, Hanks' balanced salt solution; HRP, Horseradish peroxidase; MALDI-TOF, Matrix-assisted laser desorption/ionization time of flight; MS/MS, Tandem mass spectrometry; PMN, Polymorphonuclear neutrophils; RB, Relaxation buffer

## Authors' contributions

This work was carried out equally in WMN and Gibson laboratories. RI and DJ contributed equally to this work and share first authorship. WMN and BWG likewise contributed equally to the work and share senior authorship. RI isolated PMN, separated PMN subcellular fractions, separated PMN proteins by SDS-PAGE, and performed immunoblotting. FFE was performed by KL. D B-V dB carried out the probing of subcellular fractions for dysferlin and KC provided oversight for the assessment of dysferlin content in PMN. Experiments were designed and interpreted by WMN. DJ did all the proteomic work.

## Supplementary Material

Additional file 1 Distribution of gp91^*phox *^in resting PMN. Figure A1 shows distribution of gp91^*phox *^in resting PMN. Click here for file

Additional file 2 Peptides identified by LC-MS/MS. Table A1 gives the list of polymorphonuclear neutrophil (PMN) proteins/peptides identified by mass spectrometry from fractions enriched for (A) secretory vesicles and (B) plasma membrane vesicles. Click here for file

Additional file 3 Spectral counts. Table A2 showing Spectral counts of common protein between fractions enriched for secretory vesicles (SV) and plasma membrane vesicles (PMV). Click here for file
