# Supplementary material for: Proteomic analysis of plasma membrane and secretory vesicles from human neutrophils
Source: Proteome Sci. 2007 Aug 10;5:12. doi: 10.1186/1477-5956-5-12 (PMC2075486; doi:10.1186/1477-5956-5-12)
Supplement: Additional file 1 — Distribution of gp91phox in resting PMN. Figure A1 shows distribution of gp91phox in resting PMN. [file 1477-5956-5-12-S1.doc]

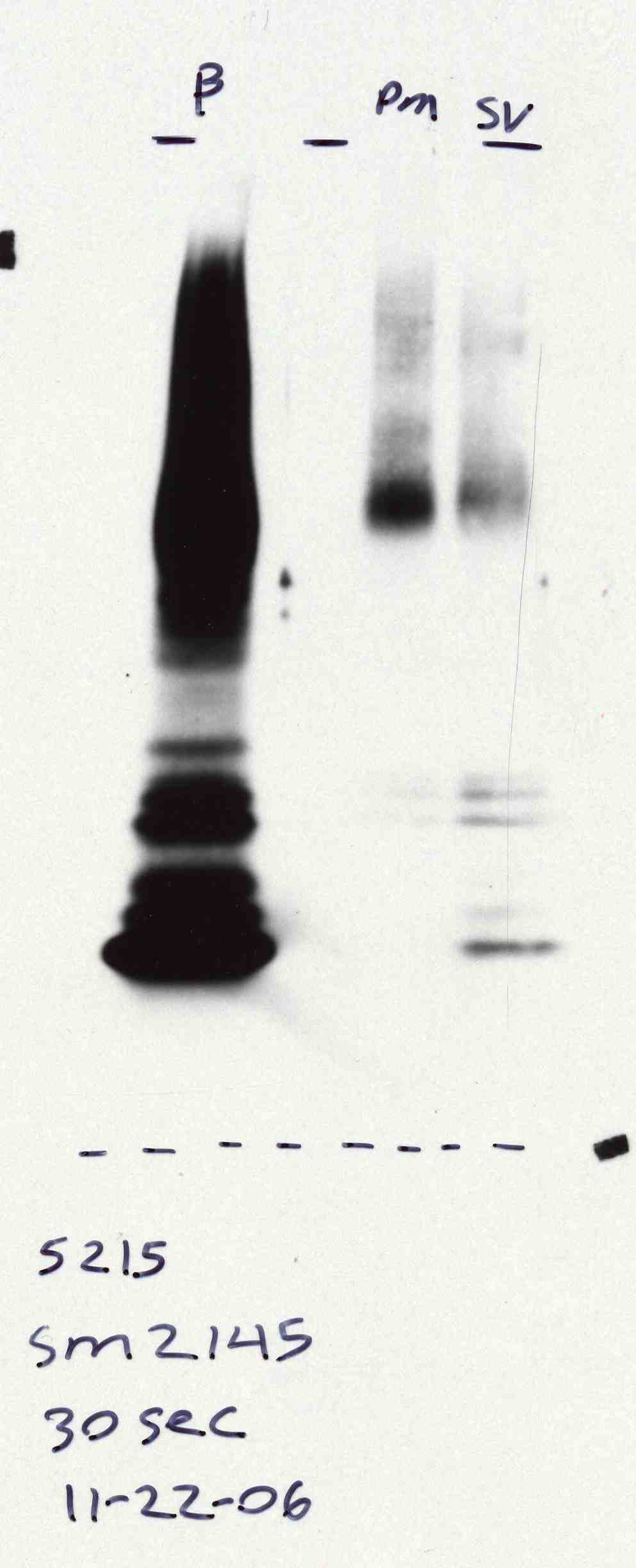

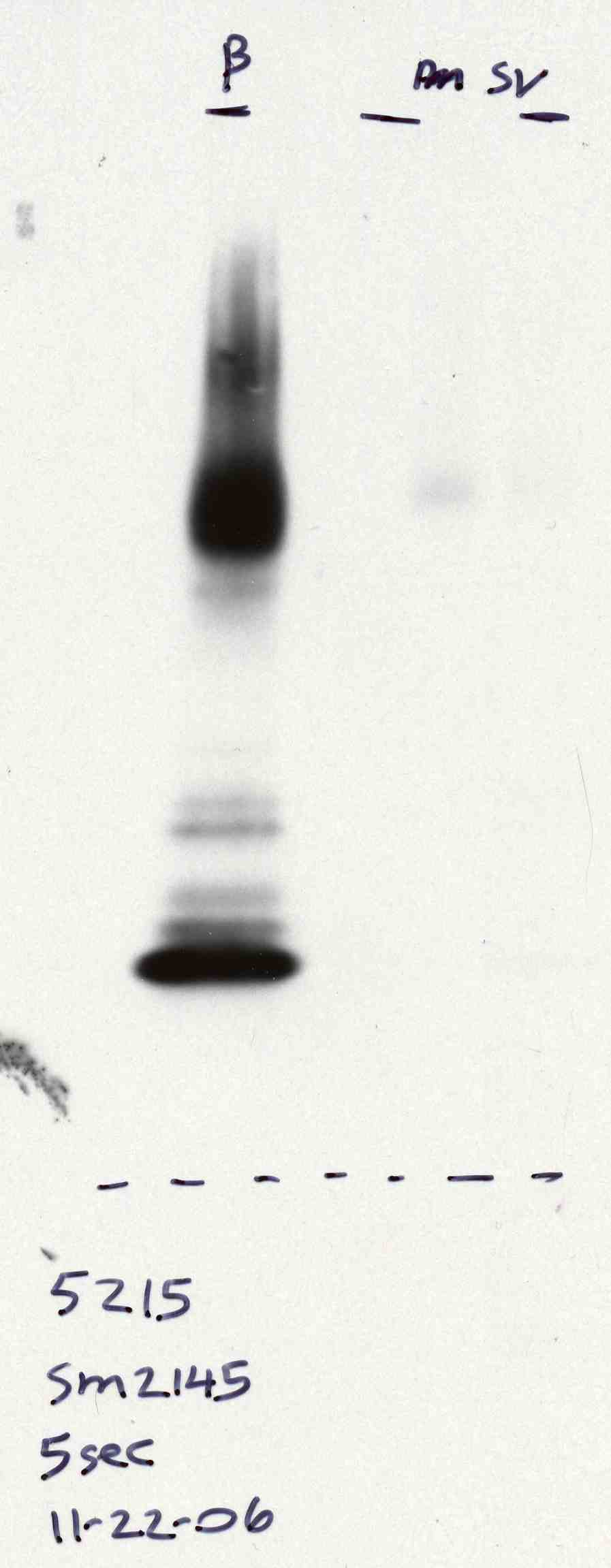


5 sec

30 sec

SG

PMV

SV

SG

PMV

SV

Distribution of gp91*^phox^* in resting PMN

**Figure A1**: An equal number of cell equivalents of specific granules (SG), PMV, and SV, were separated by SDS-PAGE, electroblotted, and probed with 54.1, a murine monoclonal antibody that recognizes gp91*^phox^*, the heavy subunit of flavocytochrome b_558_. Two exposures of the immunoblot are shown to allow an appreciation of the relative distribution of gp91*^phox^* in the subcellular fractions. Consistent with previous reports, the majority of gp91*^phox^* was detected in specific granules (~80%), with PMV and SV expressing the remaining ~20% of the total gp91*^phox^*. Proteomic analysis confirmed gp91*^phox^* in SV, although the presence of this protein in the PMV-enriched fraction was below the level needed for unambiguous identification (note: close inspection of the MS data sets indicated that 2 of the peptides identified by tandem MS in the SV fraction were likely present in PMV as well, but were below the threshold for MS/MS selection and therefore not confirmed). These data demonstrate that the PMN used in our studies were genuinely in the resting state.
