# Supplementary material for: Proteomic analysis of plasma membrane and secretory vesicles from human neutrophils
Source: Proteome Sci. 2007 Aug 10;5:12. doi: 10.1186/1477-5956-5-12 (PMC2075486; doi:10.1186/1477-5956-5-12)
Supplement: Additional file 2 — Peptides identified by LC-MS/MS. Table A1 gives the list of polymorphonuclear neutrophil (PMN) proteins/peptides identified by mass spectrometry from fractions enriched for (A) secretory vesicles and (B) plasma membrane vesicles. [file 1477-5956-5-12-S2.doc]

**TABLE A1. Mass spectrometry identification of polymorphonuclear neutrophil (PMN) proteins from fractions enriched for (A) secretory vesicles and (B) plasma membrane vesicles.**

| A. Proteins identified from PMN secretory vesicles | | | | | | |
| --- | --- | --- | --- | --- | --- | --- |
| **Gel slice no(s).** | **Protein name(s)** | **SwissProt accession number** | **MW**  **(X 10^-3^)** | **Peptides counts/**  ***Spectral counts*/**  **Sequence coverage (%)** | Peptide sequence(s) | Mascot scores |
| 1(1-15) | Integrin beta-2 (CD18 antigen) | P05107 | 82.6 | 9  *11*  (12%) | R.IGFGSFVDK.T  K.ECQPPFAFR.H  K.LGAILTPNDGR.C  K.LAENNIQPIFAVTSR.M  K.LTEIIPK.S  K.VTYDSFCSNGVTHR.N  K.GFLECGICR.C  K.YISCAECLK.F  R.YLIYVDESR.E | 52  27  29  53  37  6  35  16  23 |
| 1 (2-10) | Myosin-9 | P35579 | 226.4 | 4  *9*  (3%) | K.ASITALEAK.I  R.LEVNLQAMK.A + Oxid. (M)  K.ALELDSNLYR.I  R.IMGIPEEEQMGLLR.V + 2 Oxid. (M) | 37  37  74  43 |
| 1(2-4) | Leukocyte common antigen (CD45) | P08575 | 144.6 | 6  *9*  (5%) | K.TLILDVPPGVEK.F  R.FQCGNMIFDNK.E  R.LFLAEFQSIPR.V  R.YVDILPYDYNR.V  R.AFGDVVVK.I  K.ELISMIQVVK.Q | 39  40  47  20  29  37 |
| 1 | Erythrocyte band 7 integral membrane protein (Stomatin) | P27105 | 31.6 | 5  *6*  (18%) | K.LPVQLQR.A  R.LLAQTTLR.N  K.NLSQILSDR.E  R.YLQTLTTIAAEK.N  K.EASMVITESPAALQLR.Y + Oxid. (M) | 43  40  46  55  42 |
| 1 | Cytochrome B-245 heavy chain (GP91-PHOX) | P04839 | 65.2 | 2  *7*  (8%) | K.LLGSALALAR.A  K.MEVGQYIFVK.C + Oxid. (M) | 76  34 |
| 1 | Solute carrier family 2, facilitated glucose transporter, member 3 | P11169 | 53.9. | 2  *4*  (4%) | K.SVEMLILGR.L + Oxid. (M)  K.QVTVLELFR.V | 55  40 |
| 1 | Myeloid-associated differentiation marker (SB135) | Q96S97 | 35.3 | 2  *2*  (10%) | R.ALTQPLGLLR.L  R.TTITTTTTSSSGLGSPMIVGSPR.A + Oxid. (M) | 45  57 |
| 1 | 5-lipoxygenase activating protein (FLAP) | P20292 | 18.2 | 2  *3*  (17%) | R.TGTLAFER.V  K.YFVGYLGER.T | 34  38 |
| 1 | Leukocyte surface antigen (CD47 antigen) | Q08722 | 33.5 | 2  **3**  (6%) | K.STVPTDFSSAK.I  K.IEVSQLLK.G | 41  20  39 |
| 1 | Pantophysin | Q16563 | 28.6 | 1  *3*  (8%) | K.TVTATFGYPFR.L | 72 |
| 1 | Cytochrome P450 4F2 | P78329 | 59.9 | 1  *2*  (5%) | R.TLPSQGVDDFLQAK.A | 53 |
| 1 | *C5a anaphylatoxin chemotactic receptor* | P21730 | 39.3 | 1  *1*  (2%) | R.NVLTEESVVR.E | 66 |
| 1 | *Sarcoplasmic/*  *endoplasmic reticulum calcium ATPase 3* | Q93084 | 113.9 | 1  *1*  (1%) | K.NMLFSGTNITSGK.A + Oxid. (M) | 51 |
| 2 (1-12) | Integrin alpha-M (CD11b antigen) | P11215 | 125.5 | 16  *47*  (22%) | K.AIMEFNPR.E + Oxid. (M)  R.VQSLVLGAPR.Y  K.ILVVITDGEK.F  R.YVIGVGDAFR.S  K.TEFQLELPVK.Y  R.SLVKPITQLLGR.  R.GFGQSVVQLQGSR.V  R.LPSHSDFLAELR.K  R.VVVGAPQEIVAANQR.G  K.LTDVAIGAPGEEDNR.G  R.DHVFQVNNFEALK.T  K.SRQELNTIASKPPR.D  K.YAVYMVVTSHGVSTK.Y + Oxid. (M)  K.TLFSLMQYSEEFR.I + Oxid. (M)  K.FGDPLGYEDVIPEADR.E  R.GAVYLFHGTSGSGISPSHSQR.I | 42  63  50  45  61  51  71  59  45  46  59  38  54  64  72  42 |
| 4 | Aminopeptidase N (CD13 antigen) | P15144 | 109.4 | 5  *7*  (6%) | K.DLTALSNMLPK.G + Oxid. (M)  K.DLMVLNDVYR.V + Oxid. (M)  R.MLSSFLSEDVFK.Q + Oxid. (M)  R.AQIINDAFNLASAHK.V  R.ENSLLFDPLSSSSSNK.E | 49  35  53  38  36 |
| 6 (7-14) | Matrix metalloproteinase-9 | P14780 | 66.6 | 17  *20*  (22%) | R.QSTLVLFPGDLR.T  R.QLAEEYLYR.Y  K.SLGPALLLLQK.Q  K.QLSLPETGELDSATLK.A  R.CGVPDLGR.F  R.FQTFEGDLK.W  R.AVIDDAFAR.A  R.FGNADGAACHFPFIFEGR.S  K.LFGFCPTR.A  R.FTEGPPLHK.D  R.GSRPQGPFLIADK.W  R.KLDSVFEEPLSK.K  K.LDSVFEEPLSK.K  K.LFFFSGR.Q  K.LGLGADVAQVTGALR.S  K.MLLFSGR.R  R.MFPGVPLDTHDVFQYR.E | 49  34  59  50  15  30  45  38  52  37  28  35  58  33  82  20  30 |
| 6 (7-15) | Lactoferrin | P02788 | 76.3 | 13  *16*  (11%) | R.DSPIQCIQAIAENR.A  R.DSPIQCIQAIAENR.A  K.GGSFQLNELQGLK.S  R.DGAGDVAFIR.E  R.ESTVFEDLSDEAER.D  K.FQLFGSPSGQK.D  K.DLLFK.D  R.YYGYTGAFR.C  R.CLAENAGDVAFVK.D  K.LADFALLCLDGK.R  K.NLLFNDNTECLAR.L  K.YLGPQYVAGITNLK.K  K.YLGPQYVAGITNLKK.C | 38  52  61  58  57  37  19  22  75  71  55  60  25 |
| 9 (10-15) | Serum albumin | P02768 | 66.5 | 15  *31*  (29%) | R.FKDLGEENFK.A  K.LVNEVTEFAK.T  K.SLHTLFGDK.L  K.LCTVATLR.E  K.QEPERNECFLQHK.D  K.YLYEIAR.R  K.DVFLGMFLYEYAR.R  R.RHPDYSVVLLLR.L  K.QNCELFEQLGEYK.F  K.FQNALLVR.Y  K.KVPQVSTPTLVEVSR.N  R.RPCFSALEVDETYVPK.E  K.KQTALVELVK.H  K.AVMDDFAAFVEK.C  K.LVAASQAALGL.- | 29  51  28  33  15  40  55  51  64  51  37  60  55  73  68 |
| 9 | Dysferlin | O75923 | 237.3 | 3  *3*  (1%) | K.FFASIGER.E  K.IGETVVDLENR.L  R.DVILDDLSLTGEK.M | 18  62  72 |
| 9 | *Tyrosine-protein phosphatase non-receptor type substrate 1* | P78324 | 51.9 | 1  *1*  (2%) | R.VPPTLEVTQQPVR.A | 64 |
| 10 (9-15) | Myeloperoxidase | P05164 | 78.9 | 17  *24*  (20%) | K.NQADCIPFFR.S  R.IPCFLAGDTR.S  R.WDGERLYQEAR.K  R.KIVGAMVQIITYR.D  K.IVGAMVQIITYR.D  R.DYLPLVLGPTAMR.K  R.IANVFTNAFR.Y  R.YGHTLIQPFMFR.L  R.VVLEGGIDPILR.G  R.QNQIAVDEIR.E  R.LFEQVMR.I  R.IGLDLPALNMQR.S  R.FCGLPQPETVGQLGTVLR.N  R.VGPLLACIIGTQFR.K  R.QALAQISLPR.I  R.IICDNTGITTVSK.N  K.NNIFMSNSYPR.D | 43  49  1  38  86  40  67  23  50  61  46  45  29  76  49  78  79 |
| 10 | 60 kDa heat shock protein, mitochondrial (Hsp60) | P10809 | 57.9 | 6  *7*  (16%) | K.IGIEIIK.R  K.LSDGVAVLK.V  K.VGLQVVAVK.A  R.GYISPYFINTSK.G  K.TLNDELEIIEGMK.F + Oxid. (M)  K.ISSIQSIVPALEIANAHR.K | 44  50  66  50  89  69 |
| 10 | Alpha-1-antitrypsin | P01009 | 44.3 | 4  *5*  (14%) | K.LSITGTYDLK.S  K.LQHLENELTHDIITK.F  K.VFSNGADLSGVTEEAPLK.L  K.DTEEEDFHVDQVTTVK.V | 53  45  91  48 |
| 10 | Protein disulfide-isomerase | P07237 | 55.3 | 3  *3*  (5%) | K.ENLLDFIK.H  R.ILEFFGLK.K  R.LITLEEEMTK.Y | 26  25  30 |
| 10 | ERO1-like protein alpha | Q96HE7 | 51.9 | 1  *1*  (2%) | R.LGAVDESLSEETQK.A | 57 |
| 11 (10-14) | Eosinophil peroxidase | P11678 | 53.4 | 11  *13*  (14%) | R.DCIPFFR.S  R.IPCFLAGDTR.S  K.IMGAMVQIITYR.D +2 Oxid. (M)  R.DFLPLVLGK.A  R.VANVFTLAFR.F  R.FGHTMLQPFMFR.L + 2 Oxid. (M)  R.IVYEGGIDPILR.G  R.QDAMLVDELR.D + Oxid. (M)  R.QDAMLVDELRDR.L + Oxid. (M)  R.IGLDLAALNMQR.S + Oxid. (M)  R.IICDNTGITTVSR.D | 15  76  55  23  73  10  45  35  12  54  71 |
| 11 (15) | ATP synthase beta chain, mitochondrial | P06576 | 51.8 | 6  *7*  (15%) | K.IGLFGGAGVGK.T  K.IPVGPETLGR.I  R.TIAMDGTEGLVR.G + Oxid. (M)  R.IMNVIGEPIDER.G + Oxid. (M)  R.FTQAGSEVSALLGR.I  R.LVLEVAQHLGESTVR.T | 45  48  54  60  85  53 |
| 11 | ATP synthase alpha chain, mitochondrial | P25705 | 55.2 | 4  *5*  (7%) | R.VLSIGDGIAR.V  K.AVDSLVPIGR.G  R.VVDALGNAIDGK.G  K.TSIAIDTIINQK.R | 34  33  64  49 |
| 11 | *Ig gamma-1 chain C region* | P01857 | 36.1 | 2  *2*  (3%) | K.GPSVFPLAPSSK.S  K.STSGGTAALGCLVK.D | 39  79 |
| 11 | Aldehyde dehydrogenase 3B2 | P48448 | 42.7 | 1  *1*  (2%) | R.LLPALQSTITR.F | 57 |
| 12 | Sulfide: quinone oxidoreductase, mitochondrial | Q9Y6N5 | 49.9 | 8  *9*  (18%) | R.KVGAENVAIVEPSER.H  K.VGAENVAIVEPSER.H  K.EGNAIFTFPNTPVK.C  K.IMYLSEAYFR.K+ Oxid. (M)  K.YADALQEIIQER.N  K.TAAAVAAQSGILDR.T  R.VILAEFDYK.A  K.LFHLGMS.- + Oxid. (M) | 30  53  30  39  74  65  36  18 |
| 12 (1-18) | Actin, cytoplasmic 1 | P60709 | 41.7 | 6  *10*  (20%) | R.HQGVMVGMGQK.D  R.GILTLK.Y  R.DLTDYLMK.I  R.GYSFTTTAER.E  K.SYELPDGQVITIGNER.F  K.EITALAPSTMK.I | 2  29  28  39  44  35 |
| 12 | Citrate synthase, mitochondrial | O75390 | 49.0 | 4  *6*  (11%) | K.DILADLIPK.E  K.TVVGQITVDMMYGGMR.G  K.GLVYETSVLDPDEGIR.F  K.IVPNVLLEQGK.A | 55  21  17  45 |
| 12 | Trifunctional enzyme beta subunit, mitochondrial | P55084 | 47.5 | 3  *4*  (5%) | K.VGLPPLEK.F  R.LAAAFAVSR.L  R.TPFLLSGTSYK.D | 32  59  39 |
| 12 | Adipocyte plasma membrane-associated protein | Q9HDC9 | 46.5 | 3  *4*  (10%) | K.VLLDQLR.F  R.LLEYDTVTR.E  K.LLLSSETPIEGK.N | 37  38  42 |
| 12 | ADP-ribosyl cyclase 2 (CD157 antigen) | Q10588 | 30.1 | 2  *2*  (7%) | K.GFFADYEIPNLQK.E  R.FMPLSDVLYGR.V + Oxid. M | 64  28 |
| 12 | Dolichyl-diphosphooligosaccharide--protein glycosyltransferase 48 kDa subunit | P39656 | 46.1 | 2  *2*  (4%) | R.TLVLLDNLNVR.E  R.GFELTFK.T | 49  13 |
| 12 | *Tubulin alpha-ubiquitous chain* | P68363 | 50.2 | 1  *1*  (5%) | K.EIIDLVLDR.I | 52 |
| 12 | *Isocitrate dehydrogenase [NADP], mitochondrial* | P48735 | 46.6 | 1  *1*  (2%) | R.LIDDMVAQVLK.S + Oxid. (M) | 45 |
| 15 | Dehydrogenase/  reductase SDR family member 7 | Q9Y394 | 34.9 | 4  *5*  (17%) | R.GFFNGLR.T  K.LGVSLVLSAR.R  R.LMLISMANDLK.E + 2 Oxid. (M)  R.IDILVNNGGMSQR.S + Oxid. (M) | 35  56  42  47 |
| 15 | Guanine nucleotide-binding protein G(i), alpha-2 subunit | P04899 | 40.3 | 3  *4*  (11%) | K.LLLLGAGESGK.S  K.AMGNLQIDFADPSR.A + Oxid. (M)  R.IAQSDYIPTQQDVLR.T | 49  83  32 |
| 15 | Vacuolar ATP synthase subunit d | P61421 | 40.3 | 2  *2*  (5%) | K.LLFEGAGSNPGDK.T  R.SIAELVPK.C | 49  24 |
| 18 | Malate dehydrogenase, mitochondrial | P40926 | 33.0 | 3  *4*  (9%) | K.MISDAIPELK.A + Oxid. (M)  K.IFGVTTLDIVR.A  R.FVFSLVDAMNGK.E + Oxid. (M) | 48  35  46 |

| B. Proteins identified from PMN plasma membrane vesicles | | | | | | |
| --- | --- | --- | --- | --- | --- | --- |
| **Gel slice no(s)** | **Protein(s)** | **SwissProt accession**  **number** | **MW**  **(X10^-3^)** | **Peptides counts/**  ***Spectral counts*/**  **Sequence coverage (%)** | Peptide sequence(s) | Mascot scores |
| 5 | Integrin alpha-M  (CD11b antigen) | P11215 | 125.5 | 2  *3*  (1%) | R.VQSLVLGAPR.Y  K.ILVVITDGEK.F | 64  38 |
| 6 | Integrin alpha-IIb  (CD41 antigen) | P08514 | 110.0 | 5  *6*  (6%) | K.IVLLDVPVR.A  R.ALSNVEGFER.L  R.NVGSQTLQTFK.A  R.FGSAIAPLGDLDR.D  R.HDLLVGAPLYMESR.A + Oxid. (M) | 50  68  61  36  35 |
| 6 | Intercellular adhesion molecule-3  (ICAM-3) | P32942 | 56.3 | 2  *2*  (3%) | K.IALETSLSK.E  R.TSLTVVLLR.W | 35  62 |
| 7 | Integrin beta-2 (CD 18 antigen) | P05107 | 82.6 | 7  *8*  (9%) | K.LGGDLLR.A  R.IGFGSFVDK.T  K.ECQPPFAFR.H  K.LGAILTPNDGR.C  K.LAENNIQPIFAVTSR.M  R.RVECSGR.G  R.YLIYVDESR.E | 66  55  31  24  53  5  44 |
| 7 | Alpha-actinin 1 | P12814 | 103.1 | 6  *7*  (9%) | K.ALDFIASK.G  R.IMSIVDPNR.L + Oxid. (M)  R.LAILGIHNEVSK.I  R.TINEVENQILTR.D  K.AGTQIENIEEDFR.D  R.FAIQDISVEETSAK.E  K.MLDAEDIVGTARPDEK.A + Oxid. (M) | 35  37  42  86  63  71  42 |
| 7 | Alpha-actinin 4 | O43707 | 104.9 | 5  *6*  (8%) | K.ALDFIASK.G  R.TINEVENQILTR.D  K.LVSIGAEEIVDGNAK.M  R.FAIQDISVEETSAK.E  K.MLDAEDIVNTARPDEK.A + Oxid. (M) | 35  86  101  71  38 |
| 7 | Matrix metalloproteinase-9 | P14780 | 66.6 | 3  *4*  (4%) | R.AVIDDAFAR.A  K.SLGPALLLLQK.Q  K.LDSVFEEPLSK.K | 46  66  49 |
| 7 | *Phagocytic glycoprotein* I *(CD44 antigen)* | P16070 | 79.2 | 1/ (1%) | K.LVINSGNGAVEDR.K | 71 |
| 8 | Lactoferrin | P02788 | 76.3 | 2  *2*  (3%) | K.FQLFGSPSGQK.D  K.GGSFQLNELQGLK.S | 37  60 |
| 9 | Moesin | P26038 | 67.7 | 5  *7*  (10%) | R.EDAVLEYLK.I  K.APDFVFYAPR.L  K.KAPDFVFYAPR.L  K.TQEQLALEMAELTAR.I + Oxid. (M)  K.KTQEQLALEMAELTAR.I + Oxid. (M) | 40  55  40  68  49 |
| 11 (12-15) | Myeloperoxidase | P05164 | 78.9 | 2  *3*  (3%) | R.VVLEGGIDPILR.G  R.IGLDLPALNMQR.S + Oxid. (M) | 50  30 |
| 11 | Coronin-1A | P31146 | 51.0 | 1  *1*  (2%) | R.DAGPLLISLK.D | 55 |
| 12 | Serum albumin | P02768 | 66.5 | 1/  *1*  (4%) | K.VPQVSTPTLVEVSR.N | 44 |
| 14 (7-25) | Actin, cytoplasmic 1 | P60709 | 42.0 | 9  *15*  (21%) | R.DLTDYLMK.I + Oxid. (M)  R.GYSFTTTAER.E  K.EITALAPSTMK.I + Oxid. (M)  R.AVFPSIVGRPR.H  K.QEYDESGPSIVHR.K  K.SYELPDGQVITIGNER.F  R.VAPEEHPVLLTEAPLNPK.A  K.DLYANTVLSGGTTMYPGIADR.M + Oxid. (M)  R.KDLYANTVLSGGTTMYPGIADR.M + Oxid. (M) | 30  64  36  38  49  53  27  69  35 |
| 14 | *HLA class I histocompatibility antigen, A-26 alpha chain* | P30450 | 38.6 | 1  *1*  (10%) | R.FIAVGYVDDTQFVR.F | 81 |
| 14 | Actin-like protein 2 | P61160 | 44.8 | 1  *2*  (6%) | K.DLMVGDEASELR.S + Oxid. (M) | 52 |
| 15 (14) | Guanine nucleotide-binding protein G(i), alpha-2 subunit | P04899 | 40.3 | 4  *5*  (18%) | K.LLLLGAGESGK.S  K.TTGIVETHFTFK.D  K.AMGNLQIDFADPSR.A + Oxid. (M)  R.IAQSDYIPTQQDVLR.T | 49  32  83  32 |
| 17 (19) | Tropomyosin alpha 3 chain | P06753 | 32.8 | 3  *4*  (11%) | K.MELQEIQLK.E + Oxid. (M)  R.IQLVEEELDR.A  K.AADAEAEVASLNR.R | 51  61  48 |
| 17 | Tropomyosin beta chain | P07951 | 32.9 | 2  *3*  (7%) | K.LVILEGELER.S  R.IQLVEEELDR.A | 54  61 |
| 17 (19) | *Chloride intracellular channel protein 1* | O00299 | 26.8 | 1  *1*  (5%) | K.GVTFNVTTVDTK.R | 79 |
| 19 (25) | 14-3-3 protein zeta/delta | P63104 | 27.7 | 3  *4*  (13%) | R.NLLSVAYK.N  K.FLIPNASQAESK.V  K.SVTEQGAELSNEER.N | 38  29  96 |
| 19 | *Guanine nucleotide-binding protein G(I)/G(S)/G(T) beta subunit 1* | P62873 | 37.2 | 1  *2*  (5%) | R.LFVSGACDASAK.L | 63 |
| 19 | *Tetratricopeptide repeat protein 10* | Q13099 | 93.1 | 1  *1*  (1%) | R.MALDQVPSVNK.Q + Oxid. (M) | 55 |
| 19 | Azurocidin | P20160 | 26.9 | 1  *1*  5%) | R.QFPFLASIQNQGR.H | 44 |
| 19 | Protein FAM49B | Q9NUQ9 | 36.7 | 1  *1*  (5%) | R.INNVPAEGENEVNNELANR.M | 41 |
| 20 | Ras-related protein Rab-5A | P20339 | 23.7 | 1  *1*  (5%) | K.LVLLGESAVGK.S | 53 |
| 21 | Cathepsin G | P08311 | 28.8 | 2  *3*  (8%) | R.NVNPVALPR.A  R.TIQNDIMLLQLSR.R + Oxid. (M) | 43  53 |
| 21 | Ras-related protein Rab-27B | O00194 | 24.5 | 1  *1*  (4%) | K.FITTVGIDFR.E | 55 |
| 21 | *B-cell receptor-associated protein 31* | P51572 | 27.9 | 1  *1*  (3%) | K.LDVGNAEVK.L | 46 |
| 21 | *Synaptosomal-associated protein 23* | O00161 | 23.4 | 1  *1*  (4%) | R.IEEGLDQINK.D | 45 |
| 22 | Ras-related protein Rap-1b | P61224 | 20.8 | 3  *4*  (18%) | K.LVVLGSGGVGK.S  K.YDPTIEDSYR.K  K.DTDDVPMILVGNK.C + Oxid. (M) | 43  35  56 |
| 22 | Ras-related protein Rap-1A | P62834 | 20.9 | 3  *4*  (18%) | K.LVVLGSGGVGK.S  K.YDPTIEDSYR.K  K.DTEDVPMILVGNK.C + Oxid. (M) | 43  35  38 |
| 23 (24) | Myosin regulatory light chain 2, nonsarcomeric | P19105 | 19.7 | 2  *3*  (12%) | K.LNGTDPEDVIR.N  K.EAFNMIDQNR.D + Oxid. (M) | 44  35 |
| 23 | Cofilin, non-muscle isoform | P23528 | 18.4 | 1  *2*  (11%) | R.YALYDATYETK.E | 73 |
| 24 | Golgi-associated plant pathogenesis-related protein 1 | Q9H4G4 | 17.1 | 2  *3*  (16%) | K.ASASDGSSFVVAR.Y  R.EAQQYSEALASTR.I | 84  68 |
| 24 | Myosin light polypeptide 6 | P60660 | 16.8 | 2/  *3*  (14%) | K.EAFQLFDR.T  R.ALGQNPTNAEVLK.V | 40  49 |
| 24 | *Interferon-induced transmembrane protein 1* | P13164 | 13.9 | 1  *1*  (12%) | K.MVGDVTGAQAYASTAK.C + Oxid. (M) | 86 |

**TABLE S1: Major proteins identified from fractions enriched for secretory (A) and plasma membrane (B) vesicles by** **nano HPLC electrospray ionization MS/MS.**

The γ fraction of resting PMN were resolved into secretory vesicles and plasma membrane vesicles using free-flow electrophoresis were separated on 4-20% SDS-PAGE and visualized by SYPRO ruby staining as shown in Figure 2A. The bands (indicated by numbers) from top to bottom in each lane were excised from the gel with the help of band picker, 1.5 mm and analyzed by using an Agilent 1100 Series nanoflow liquid chromatograph (LC) system coupled to an Agilent 1100 Series XCT ion trap mass spectrometer. Proteins identifications were made after searching all MS/MS spectra associated with a single gel band against the SwissProt database using the Mascot search engine. Proteins that had two or more peptide hits and a Mascot score of >34 were considered valid identifications. Proteins with only one hit were validated according to criteria listed by (reference 61 in the manuscript), with the restraint that they represent a unique peptide in the human proteome as confirmed by BLAST searches and also validated by “The GPM” site, which consist of large MS/MS spectra database. To remove all weak matches we have set off the threshold at 0.05 in the ion-score cut-off field during mascot search. Also, the single hit peptide spectra were manual inspected using the following additional criteria: good signal-to-noise, coherent y or b-ion series and y-ion with N-proline must be intense. Proteins that survived this criteria are listed in italics and were primarily represented among proteins with a molecular weight of < 30 kDa, where the probability of multiple peptide hits would be expected to be at its the lowest. All proteins listed with a single peptide hit were also validated by the GPM website, <http://www.thegpm.org/GPM/index.html>. The protein was added in the list only if its peptide had been previously identified in the GPM database with >0.5 cos (θ) compared against the best MS/MS spectra available. The protein names, Swiss-Prot protein accession numbers, theoretical masses, peptide counts (non-redundant number of peptides identified from a protein), spectral counts (total number of MS/MS spectra identified from a protein), sequence coverage (percentage of a protein’s amino acid sequence covered by peptide identified by mass spectrometry), peptide sequence and mascot scores identified in secretory vesicles (A) and plasma membrane vesicles (B) are listed.
