# Supplementary material for: Proteomic analysis of plasma membrane and secretory vesicles from human neutrophils
Source: Proteome Sci. 2007 Aug 10;5:12. doi: 10.1186/1477-5956-5-12 (PMC2075486; doi:10.1186/1477-5956-5-12)
Supplement: Additional file 3 — Spectral counts. Table A2 showing Spectral counts of common protein between fractions enriched for secretory vesicles (SV) and plasma membrane vesicles (PMV). [file 1477-5956-5-12-S3.doc]

**Table A2. Spectral counts of common proteins between fractions enriched**

**for secretory vesicles (SV) and plasma membrane vesicles (PMV).**

| **Protein name(s** | **Secretory vesicles** | **Plasma membrane vesicles** |
| --- | --- | --- |
| Integrin beta 2, CD 18 antigen (P05107) | 11 | 8 |
| Integrin alpha-M, CD11b antigen (P11215) | 47 | 3 |
| Matrix Metalloproteinase-9 (P14780) | 20 | 4 |
| Lactoferrin (P02788) | 16 | 2 |
| Serum Albumin (P02768) | 31 | 1 |
| Myeloperoxidase (P05164) | 24 | 3 |
| Actin, Cytoplasmic I (P60709) | 10 | 15 |
| Guanine nucleotide binding protein G (i) alpha2 subunit (P04899) | 4 | 5 |

**Table A2: Common protein and their spectral counts identified by HPLC-MS/MS between fractions enriched for secretory vesicles (SV) and Plasma membrane vesicles (PMV).** Based on densitometry comparisons of the two gel lanes, the total protein loaded on the secretory vesicles was approximately 2.6 fold higher that of the plasma membrane vesicles. In addition, the total spectra count of all peptides assigned to these two vesicle populations was consistent with this estimate of total proteins, i.e., total spectral counts for plasma membrane vesicles (118) and secretory vesicles (307). Therefore, one needs to consider this loading difference when making relative protein abundances comparisons between these two vesicle compartments. See main text.
